# Antimicrobial Resistance and Virulence of Non-Typhoidal *Salmonella* from Retail Foods Marketed in Bangkok, Thailand

**DOI:** 10.3390/foods11050661

**Published:** 2022-02-24

**Authors:** Thida Kong-Ngoen, Sirijan Santajit, Witawat Tunyong, Pornpan Pumirat, Nitat Sookrung, Wanpen Chaicumpa, Nitaya Indrawattana

**Affiliations:** 1 Department of Microbiology and Immunology, Faculty of Tropical Medicine, Mahidol University, Bangkok 10400, Thailand; thida.kon@mahidol.ac.th (T.K.-N.); witawat.tun@mahidol.ac.th (W.T.); pornpan.pum@mahidol.ac.th (P.P.); 2 Department of Medical Technology, School of Allied Health Sciences, Walailak University, Nakhon Si Thammarat 80160, Thailand; sirijan.sa@wu.ac.th; 3 Research Center in Tropical Pathobiology, Walailak University, Nakhon Si Thammarat 80160, Thailand; 4 Biomedical Research Incubator Unit, Department of Research, Faculty of Medicine Siriraj Hospital, Mahidol University, Bangkok 10700, Thailand; nitat.soo@mahidol.ac.th; 5 Center of Research Excellence on Therapeutic Proteins and Antibody Engineering, Department of Parasitology, Faculty of Medicine Siriraj Hospital, Mahidol University, Bangkok 10700, Thailand; wanpen.cha@mahidol.ac.th

**Keywords:** food-borne salmonellosis, *Salmonella* Enteritidis, multi-drug resistance, invasion genes bacterial virulence

## Abstract

Nontyphoidal-*Salmonella* bacteria cause foodborne gastroenteritis that may lead to fatal bacteremia, osteomyelitis, and meningitis if not treated properly. The emergence of multidrug-resistant *Salmonella* strains is a global public health threat. Regular monitoring of genotypes and phenotypes of *Salmonella* isolated from humans, animals, foods, and environments is mandatory for effective reduction and control of this food-borne pathogen. In this study, antimicrobial-resistant and virulent genotypes and phenotypes of *Salmonella* isolated from retail food samples in Bangkok, Thailand, were investigated. From 252 raw food samples, 58 *Salmonella* strains that belonged only to serotype Enteritidis were isolated. Disc diffusion method showed that all isolates were still sensitive to amikacin and carbapenems. More than 30% of the isolates were resistant to ampicillin, tetracycline, and ciprofloxacin. Twenty isolates resist at least three antibiotic classes. Minimum inhibitory concentration tests showed that 12.07% of the isolates produced extended-spectrum β-Lactamase. Polymerase chain reaction indicated that 32.76, 81.03, 39.66, and 5.17% of the isolates carried *bla*_TEM-1_, *tet*A, *sul*2, and *dfr*A7, respectively. All isolates were positive for invasion-associated genes. Effective prevention and control of *Salmonella* (as well as other food-borne pathogens) is possible by increasing public awareness and applying food hygienic practices. Active and well harmonised “One Health” co-operation is required to effectively control food-borne zoonosis.

## 1. Introduction

*Salmonella* causes food-borne gastroenteritis (salmonellosis) with high and increasing prevalence worldwide [1,2,3]. The bacteria are ubiquitously present in the environment and throughout the food chain, i.e., farm-to-folk. Humans become infected through the consumption of contaminated water or foods mainly of animal origins, such as poultry meat, eggs, pork, beef, dairy products, and ready-to-eat produce [4,5]. *Salmonella* serovars with human host preference include *S.* Typhimurium and *S.* Enteritidis [6,7]. Clinical symptoms of salmonellosis usually begin 6–8 h to 7 days after infection and are characterised by abdominal cramp, fever, and diarrhoea [8]. The diseases can be self-limited in healthy individuals but may be severe, which requires prompt medical attention and may also be life-threatening if the bacteria invade beyond the gastrointestinal tract [9]. According to the World Health Organization (WHO), *Salmonella* is one of the key causative agents of diarrheal disease, which inflicts not only huge medical intervention expenses but also loss of productivity [10].

Pathogenesis of *Salmonella* is related to the abundance of the virulence genes in the chromosomally located *Salmonella* pathogenicity islands (SPIs) [11,12]. Among the virulence-associated genes are *inv*A, which encodes the type III secretion system, and the *hil*A, which encodes an OmpR/ToxR family transcriptional regulator that activates the expression of invasion genes required for *Salmonella* invasion into host intestinal epithelial cells [13,14,15]. Besides, *Salmonella* bacteria also harbour plasmids carrying a myriad of antimicrobial resistance genes, such as *bla*_TEM-1_ (class A broad-spectrum β-lactamase, TEM-1), *bla*_CMY-2_ (class C β-lactamase CMY-2), *tet*A (tetracycline efflux major facilitator superfamily (MFS) transporter, TetA), *tet*C (tetracycline resistance-associated transcriptional repressor, TetC), *sul*2 (sulfonamide-resistance gene), and *dfr*A7 (dihydrofolate reductase, a single gene cassette within the class 1 integrons). These genes contribute to drug-resistant phenotypes, which are currently the major global public health worrisome [16,17,18,19,20,21,22]. 

Antibiotic resistance among bacteria is a global phenomenon. Regular monitoring of serotypes and drug-resistant phenotypes and genotypes of *Salmonella* that contaminate foods may help track the cause of the food-borne diseases and may lead to appropriate food safety policy for intervention, prevention, and/or effective treatment measures of food-borne illnesses. Therefore, in this study, we assessed the prevalence of antimicrobial phenotypes and drug resistance-associated and virulence genes in *Salmonella* isolated from retail food samples in the Bangkok metropolitan area.

## 2. Materials and Methods

### 2.1. Sample Collection and Bacterial Isolation and Identification

Five different food categories (chicken, *n* = 44; pork and beef, *n* = 28; seafood, *n* = 60; fruits and vegetables, *n* = 60; and dairy products, *n* = 60) comprising 252 samples were collected from 19 wet markets and 2 supermarkets between October and December 2017. All markets are located in the central and peripheral districts of the Bangkok Metropolitan area. Food samples were maintained in sterile bags on ice and transferred to the laboratory within 2 h.

Food samples were processed according to the international standard, five-step method of the ISO protocol: 6579: 2002 Microbiology of Food and Animal Feeding Stuffs-Horizontal Method for the Detection of *Salmonella* spp. [23,24]. Firstly, individual samples were pre-enriched in a non-selective medium. Twenty-five grams of each sample was placed in a sterile 500 mL flask containing 225 mL of Trypticase Soy Broth and incubated at 37 °C for 18–24 h. Then, 0.1 mL of each overnight culture was inoculated into 10 mL of selective enrichment medium, Rappaport-Vassiliadis Soya broth (Merck, Darmstadt, Germany), and incubated at 42 °C for 24 h. The cultures (0.1 mL aliquots) were spread onto selective agar plates, i.e., xylose lysine deoxycholate agar (XLD) and *Salmonella*–*Shigella* agar (SS) selective plates, and the plates were incubated at 37 °C for 18–24 h. Suspected *Salmonella* colonies (small red colonies with/without central black dots on XLD agar and translucent colourless colonies with/without central black dots on SS agar) were subjected to conventional biochemical assays for *Salmonella* verification, including triple sugar iron (TSI) agar utilisation, deamination of lysine, ornithine decarboxylation, citrate and urease productions, and indole formation, as well as motility testing [25].

### 2.2. Serotyping of the Salmonella Isolates

All *Salmonella* isolates were serotyped using polyvalent O and H antisera by slide agglutination technique (Kauffmann–White–Le Minor scheme) [26]. The assay was performed according to the manufacturer’s instructions (Serosystem, Clinag, Bangkok, Thailand). Briefly, individual *Salmonella* colonies were suspended in normal saline solution on glass slides. They were mixed separately with 9 polyvalent *Salmonella* antisera reagents in a 1:1 ratio, and the slides were rocked in a circular motion for 30 s. Bacterial agglutination was visually observed. Strains giving negative or positive agglutinations were recorded.

### 2.3. Determination of Intestinal Cell Invasion by Salmonella Isolates

The ability of the isolated *Salmonella* strains to invade human colon carcinoma cells (Caco-2 cell line) was investigated. Confluent Caco-2 cell monolayer was established in 24-well tissue culture plates (approximately 2 × 10^5^ cells/well) containing Dulbecco’s modified Eagle’s medium (DMEM) (Gibco, NY, USA) supplemented with 10% fetal bovine serum and 50 µg/mL gentamicin at 37 °C in 5% CO_2_ atmosphere. The monolayers were rinsed twice in phosphate-buffered saline, pH 7.4 (PBS). Cells were infected with individual *Salmonella* strains at a multiplicity of infection (MOI) 1:50 [27]. Plates were incubated at 37 °C in 5% CO_2_ incubator for 4 h. The cells were rinsed to remove extracellular bacteria and replenished with DMEM containing gentamicin (50 μg/mL) for 1.5 h. Cells were then rinsed with PBS and stained with Giemsa reagent. *Salmonella* invasion into the Caco-2 cells was observed under inverted microscopy (200 and 400× magnifications) (Zeiss, Jena, Germany). Alternatively, the infected cells were lysed by adding 1% Triton X-100 (Sigma); the lysate was spread on an LB plate and incubated at 37 °C for 24 h. The presence of bacterial colonies on the cultured plate indicates the invasive ability of the bacterial isolate.

### 2.4. Antimicrobial Resistance Profiles

Antimicrobial susceptibility was evaluated based on Clinical and Laboratory Standards Institute 2017 (CLSI 2017) guidelines using the disc diffusion method. Briefly, *Salmonella* isolates were aerobically cultured in 10 mL of Mueller–Hinton (MH) broth (Oxoid, Hampshire, UK) at 37 °C for 24 h. Overnight cultures were adjusted to an optical density of 0.5 MacFarland units. The bacterial suspensions were aseptically spread onto MH agar plates, and the plates were allowed to dry for 2–4 min. Individual antimicrobial discs were placed on the surface using a disc dispenser, and the plates were incubated at 37 °C for 24 h. The tested antibiotics were ampicillin (10 µg), ampicillin/sulbactam (10 µg/10 µg), piperacillin/tazobactam (100 µg/10 µg), cefepime (30 µg), cefotaxime (30 µg), ceftazidime (30 µg), ceftriaxone (30 µg), gentamicin (10 µg), amikacin (30 µg), ertapenem (10 µg), meropenem (10 µg), imipenem (10 µg), tetracycline (30 µg), ciprofloxacin (5 µg), and trimethoprim/sulfamethoxazole (1.75/23.25 µg) (Oxoid). Extended-spectrum β-lactamase (ESBL) production was also determined using the combination disc test comprising ceftazidime with and without clavulanate and cefotaxime with and without clavulanate (Oxoid). A positive test was defined as a ≥5 mm difference in zone diameter between the respective two discs. The CLSI 2017 criteria were followed for the interpretation of the antimicrobial susceptibility results.

### 2.5. Polymerase Chain Reaction for Determination of Drug Resistance and Virulence Genes of the Salmonella Isolates

All *Salmonella* isolates were screened for the presence of virulence genes (*inv*A and *hil*A) and antimicrobial resistance genes (*tet*A, *tet*C, *bla*_TEM-1_, *bla*_CMY-2_, *sul*2, and *dfr*A7) by using PCR. Genomic DNA was extracted from each *Salmonella* culture using the conventional boiling method [27]. Two millilitres of each bacterial culture were centrifuged at 14,000× *g* for 5 min. Sterile distilled water (600 μL) was added to the pellet and re-centrifuged. The supernatant was discarded, and 200 µL of sterile distilled water was added to the pellet. The sample was then placed in a 100 °C heat-block for 10 min, immediately cooled on ice for 5 min, and centrifuged at 14,000× *g* for 5 min. The supernatant was used as a PCR template.

PCR was conducted using primers listed in Table 1. The PCR reaction mixture (25 μL) contained 3 μL of DNA template, 2.5 μL of 10× *Taq* buffer, 2 mM MgCl_2_, 0.2 mM dNTP, 1 μM each primer, and 1 U of *Taq* DNA polymerase (Thermo Fisher Scientific, Waltham, MA, USA). The thermal cycles were initial denaturation at 94 °C for 5 min, 35 cycles of denaturation at 94 °C for 45 s, annealing at 52–60 °C for 40 s, extension at 72 °C for 40 s and a final extension at 72 °C for 7 min. *Salmonella* Enteritidis ATCC 13076 and constructed plasmids containing the antibiotic-resistant genes served as positive controls, while buffer alone (without DNA template) served as a negative control. The PCR products were electrophoresed on 1.5% (*w*/*v*) agarose gels in 100 mL of 1× TAE buffer and stained with ethidium bromide. DNA bands were visualised using the ChemiDoc MP imaging system (Bio-Rad, Hercules, CA, USA). 

### 2.6. Statistical Analysis

The statistical analysis and data comparison were performed using one-way ANOVA in GraphPad Prism version 9 (La Jolla, CA, USA). The *p*-value < 0.05 was considered statistically significant. 

## 3. Results

### 3.1. Prevalence and Serotypes of Salmonella in Retail Food Samples 

Fifty-eight *Salmonella* isolates (23%) were recovered from a total of 252 retail food samples. All of them belonged to serovar Enteritidis. The isolated bacteria were from chicken (36 isolates, 62.07%), pork (16 isolates, 27.59%), and beef (6 isolates, 10.34%). The comparative prevalence of *S*. Enteritidis isolated from chicken and pork, chicken and beef, chicken and fruits, chicken and vegetables, pork and fruits, and pork and vegetables were different (*p* < 0.001). The *Salmonella* prevalence in pork and beef samples was also different (*p*  <  0.05). Nevertheless, no difference was found between samples of beef and fruits, beef and vegetables, and fruits and vegetables (*p* > 0.05). The isolates were further classified into six different groups, i.e., B (*n* = 17; 29.31%), C (*n* = 22; 37.93%), E (*n* = 15; 25.86%), G (*n* = 1; 1.72%), and I (*n* = 2; 3.45%), and non-A–I (*n* = 1; 1.72%). Group C was predominant in this study (Table 2).

### 3.2. Antimicrobial and Virulence Genotypes of the Salmonella Isolates

**Figure 1:**
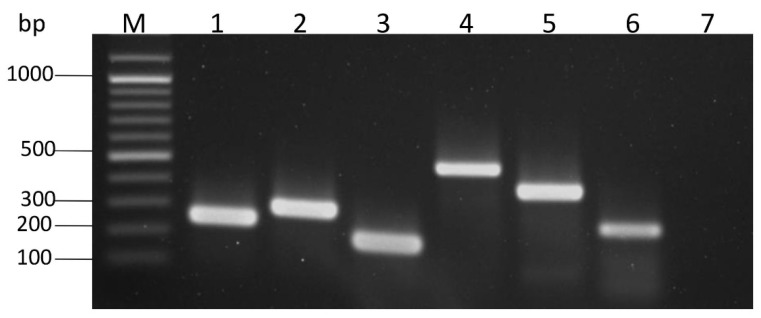
Molecular detection of virulence and drug-resistance associated genes of *Salmonella* isolates using PCR methods. Lane M: 100 bp plus DNA ladder; Lane 1: the representative *inv*A amplicon; Lane 2: the representative *hil*A amplicon; Lane 3: the representative *tet*A amplicon; Lane 4: the representative *bla*_TEM-1_ amplicon; Lane 5: the representative *sul*2 amplicon; Lane 6: the representative *dfrA*7 amplicon, and Lane 7: negative control.

PCR was used to determine drug resistance and virulence genes of the *Salmonella* isolates. The drug resistance and virulence genes that were detected included *inv*A, *hil*A, *tet*A, *bla*_TEM-1_, *sul*2, and *dfr*A7, of which their PCR amplicon sizes were 244, 296, 210, 504, 405, and 265 base pairs (bp), respectively (Figure 1). The invasion operon genes, *inv*A and *hil*A, were detected in all isolates. The *bla*_TEM-1_ (*n* = 19; 32.76%), *tet*A (*n* = 47; 81.03%), *sul*2 (*n* = 23; 39.66%) and *dfr*A7 (*n* = 3; 5.17%) genes were carried by the resistance strains, a clear difference was noticed in the occurrence of these genes among the isolates. None of the isolates was positive for *bla*_CMY-2_ and *tetC* genes. The pork and chicken isolates were positive for at least one antimicrobial resistance-associated gene. The *tet*A was the most prevalent gene among the *Salmonella* isolated from pork and chicken, followed by *sul2*. None of the beef isolates carried the antimicrobial resistance-associated gene, and all of them were not resistant to any of the antibiotics tested (Table 2).

### 3.3. Antimicrobial Phenotypes of the Salmonella Isolates

**Figure 2:**
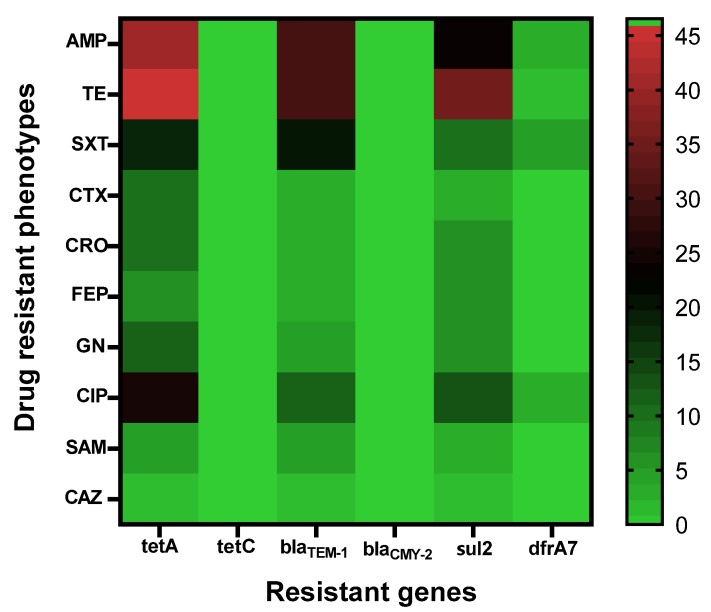
Heatmap of percent distribution for drug-resistant phenotypes and genotypes of *S.* Enteritidis isolates that were present in at least one isolate with antibiotic-resistant phenotype. The colored strip depicts the percentage of genes associated with a particular antibiotic-resistant phenotype. Created using GraphPad Prism version 9 (La Jolla, CA, USA).

Antibiotic sensitivity testing was performed for the 58 *Salmonella* isolates, and the results are shown in Table 3. All isolates were sensitive to ertapenem and amikacin. Twenty-six isolates (44.83%) were resistant to ampicillin (penicillin group); 3 isolates (5.17%) were resistant to ampicillin/sulbactam (β-lactam combination agents); 7 isolates (12.07%) each were resistant to cefepime, cefotaxime, and ceftriaxone, and 1 isolate resisted ceftazidime (cephalosporin group); 7 isolates (12.07%) resisted gentamicin (aminoglycoside group); 32 isolates (55.17%) resisted tetracycline (tetracycline group); 20 isolates (34.48%) resisted ciprofloxacin (fluoroquinolone group); and 12 isolates (20.69%) resisted trimethoprim/sulfamethoxazole (folate pathway antagonist group). Seven isolates (12.07%) were ESBL producing *S.* Enteritidis. Among 58 isolates, 20 (34.48%) were multi-drug resistant (MDR); *Salmonella* group B were resistant to at least three antibiotic classes (Table 3). A heatmap of the distribution of antimicrobial resistance genes and their phenotypes is illustrated in Figure 2. The isolates with phenotypic resistance to at least one antibiotic are displayed. 

### 3.4. Caco-2 Invasion Assay on Isolates

**Figure 3:**
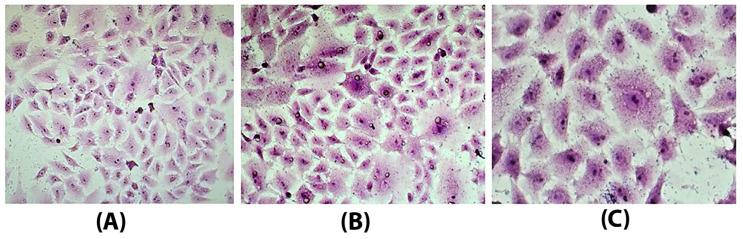
Microscopic appearance of Giemsa’s stained CaCo-2 cells: (**A**) before (**B**,**C**) and after infecting with the representative *Salmonella* Enteritidis isolate no. 44 (Sal44). Bacteria are predominantly seen in the CaCo-2 cells’ cytoplasm (original magnification 200× and 400×, respectively).

The ability of *S.* Enteritidis isolates to invade human intestinal epithelial (Caco-2) cells was determined. All 58 isolates, which carried *inv*A and *hil*A genes, could invade the Caco-2 cells. The cell invasion of the representative isolate is shown in Figure 3.

## 4. Discussion

Regular monitoring of serotypes, antimicrobial-resistant characteristics, and virulence of food-borne pathogenic bacteria, particularly *Salmonella enterica*, can provide useful epidemiological information on food-borne bacterial infections in a locality [34]. In recent decades, *S.* Enteritidis has been identified as the predominant causative agent of salmonellosis in Thailand [35,36]. In this study, 23% of the raw food samples collected from open markets in the Bangkok metropolitan region were found to be contaminated with *Salmonella*. The contaminated food samples were solely meat (chicken > pork > beef), while seafood, fruits, vegetables, and dairy products were not contaminated. All contaminated *Salmonella* isolates belonged to serovar Enteritidis, of which group C was predominant. When compared with the prevalence of *S.* Enteritidis from raw foods in other countries, e.g., abattoirs in Iran and butcher shops and supermarkets in Pakistan where the prevalence rates were 43 and 37.5%, respectively, the bacterial prevalence in our study was less [37,38]. 

Drug susceptibility testing data revealed that even though the *S.* Enteritidis isolated in this study were resistant to many groups of antibiotics, including penicillin, combined β-lactam agents, cephalosporins, aminoglycosides, tetracyclines, fluoroquinolones, and folate pathway antagonists, most of these MDR *Salmonella* strains were still sensitive to amikacin and carbapenems. Even though the isolates of this study showed high resistance to ampicillin, tetracycline, and ciprofloxacin, the prevalence of resistant isolates was still less compared to those isolated in Brazil, Iran, and China [39,40,41].

Invasion into cultured epithelial cells has been routinely used for determining *Salmonella* virulence [42,43,44,45,46]. Genotypic and phenotypic analysis of the *S.* Enteritidis isolates of this study revealed that the bacteria carried invasion genes (*inv*A and *hil*A). Nevertheless, they showed different degrees of invasiveness when tested by the invasion assay using intestinal epithelial (Caco-2) cells. The results conformed to those reported previously by others [47,48,49,50,51]. Most MDR *Salmonella* isolates were found to carry the antimicrobial-associated genes, namely, *bla*_TEM-1_, *tet*A, *sul*2, and *dfr*A7 [28,52]. The prevalence of drug resistance genes was highest for *tetA*, followed by *sul*2, *bla*_TEM-1_, and *dfr*A7. No isolate carried *tet*C and *bla*_CMY-2_. Detail analysis of the entire genomes of the isolates by using next-generation sequencing should be performed further to provide the insight information for guiding appropriate treatment decisions and allow rapid tracking of transmission of the drug-resistant clones.

Epidemics of human salmonellosis are generally associated with a particular prevalent serovar and serotype of *S. enterica*. Epidemic tracking of the bacterial pathogens, e.g., through identification of the causative strain origin as well as the antimicrobial susceptibility pattern and their virulence characteristics in an outbreak, can be readily performed either phenotypically or genotypically, or both [29]. It is also noteworthy that retail food products undergo extensive processing and handling during production, which potentially enhance the risk of contamination [30]. Appropriate food hygienic education for end-consumers must be regularly implemented. Since the majority of food-borne diseases, including salmonellosis, are zoonotic, thus, improving food hygiene through health education and “One Health” approach should be practiced at all levels, i.e., from a locale to a nation-wide and global responsible practices.

## 5. Conclusions

In conclusion, the findings of this study supported the notion of the divergence of *Salmonella* serotypes isolated from a variety of raw food samples from the opened market and hypermarket in Bangkok and its periphery, Thailand. The findings also provided insight into the molecular characterisation of virulence- and drug-resistance traits, as well as the antimicrobial susceptibility pattern of the bacterial pathogen. The spread of MDR strains of *Salmonella* isolates with the cell invasion potential was become growing continuously. This requires good planning and effective control programs to prevent and manage infections for their spreading to community and public health. 

## Figures and Tables

**Table 1:** PCR primers used for amplification of different drug resistance-associated and virulence genes.

Gene Name	Oligonucleotide Sequence (5′-3′)	Product Size (bp)	Annealing Temperature (°C)	Reference
*inv*A	Forward: ACAGTGCTCGTTTACGACCTGAAT Reverse: AGACGACTGGTACTGATCGATAAT	244	60	[28]
*hil*A	Forward: CGTGAAGGGATTATCGCAGT Reverse: GTCCGGGAATACATCTGAGC	296	56	[29]
*bla*_TEM-1_	Forward: TTGGGTGCACGAGTGGGT Reverse: TAATTGTTGCCGGGAAGC	504	56	[30]
*bla*_CMY-2_	Forward: ATAACCACCCAGTCACGC Reverse: CAGTAGCGAGACTGCGCA	631	52	[31]
*sul*2	Forward: CGGCATCGTCAACATAACC Reverse: GTGTGCGGATGAAGTCAG	405	60	[31]
*tet*A	Forward: GCTACATCCTGCTTGCCTTC Reverse: CATAGATCGCCGTGAAGAGG	210	52	[32]
*tet*C	Forward: CTTGAGAGCCTTCAACCCAG Reverse: ATGGTCGTCATCTACCTGCC	418	52	[32]
*dfr*A7	Forward: GGTAATGGCCCTGATATCCC Reverse: TGTAGATTTGACCGCCACC	265	50	[33]

**Table 2:** Serotypes, antibiotic resistance profiles, virulence genes, and drug resistance-associated genes of *Salmonella* Enteritidis isolates of this study.

*Salmonella* Isolates	Source	Antibiotic-Resistant Profile	*Salmonella* Serotype	Virulence Gene		Drug Resistance Associated Gene					
				*inv*A	*hil*A	*tet*A	*tet*C	*bla*_TEM-1_	*bla*_CMY-2_	*sul2*	*dfr*A7
Sal1	pork	AMP, TE, and SXT	*B*	+	+	+	−	+	−	+	−
Sal2	pork	AMP, TE, and SXT	*B*	+	+	+	−	+	−	+	−
Sal3	pork	AMP and SXT	*E*	+	+	+	−	+	−	+	+
Sal4	pork	AMP, CTX, CRO, FEP, GN, and TE	*E*	+	+	+	−	−	−	+	−
Sal5	pork	AMP, CTX, CRO, FEP, GN, and TE	*E*	+	+	+	−	−	−	+	−
Sal6	pork	AMP, TE, CIP, and SXT	*E*	+	+	+	−	+	−	+	+
Sal7	pork	AMP, CTX, CRO, FEP, GN, and TE	*E*	+	+	−	−	−	−	+	−
Sal8	pork	AMP and TE	*C*	+	+	+	−	+	−	+	−
Sal9	pork	−	*E*	+	+	+	−	−	−	−	−
Sal10	pork	AMP, CTX, CRO, FEP, GN, and TE	*E*	+	+	+	−	−	−	−	−
Sal11	pork	−	*E*	+	+	+	−	−	−	−	−
Sal12	pork	AMP and TE	*B*	+	+	+	−	+	−	+	−
Sal13	pork	AMP	*C*	+	+	+	−	−	−	−	−
Sal14	pork	AMP, TE, CIP, and SXT	*B*	+	+	−	−	+	−	−	−
Sal15	pork	AMP, CTX, CRO, FEP, GN, and TE	*E*	+	+	+	−	−	−	−	−
Sal16	pork	AMP, SAM, CAZ, CTX, CRO, FEP, GN, and TE	*B*	+	+	+	−	+	−	+	−
Sal17	chicken	AMP, SAM, TE, and SXT	*B*	+	+	+	−	+	−	−	−
Sal18	chicken	−	*I*	+	+	+	−	−	−	−	−
Sal20	chicken	−	*I*	+	+	+	−	−	−	−	−
Sal21	chicken	−	*C*	+	+	+	−	−	−	−	−
Sal22	chicken	−	*C*	+	+	−	−	−	−	−	−
Sal23	chicken	CIP	*C*	+	+	+	−	−	−	−	−
Sal24	chicken	CIP	*C*	+	+	+	−	−	−	−	−
Sal25	chicken	−	*E*	+	+	+	−	−	−	−	−
Sal26	chicken	TE and CIP	*B*	+	+	+	−	−	−	−	−
Sal27	chicken	CIP	*C*	+	+	+	−	−	−	−	−
Sal28	chicken	−	*C*	+	+	+	−	−	−	−	−
Sal29	chicken	−	*Non A-I*	+	+	+	−	−	−	−	−
Sal30	chicken	AMP, TE, CIP, and SXT	*B*	+	+	+	−	+	−	−	−
Sal31	chicken	AMP, TE, CIP, and SXT	*B*	+	+	+	−	+	−	−	−
Sal32	chicken	TE	*C*	+	+	+	−	−	−	+	−
Sal33	chicken	CIP	*C*	+	+	+	−	−	−	−	−
Sal34	chicken	TE and CIP	*C*	+	+	+	−	−	−	+	−
Sal35	chicken	TE and CIP	*C*	+	+	−	−	−	−	+	−
Sal36	chicken	AMP, TE, and SXT	*B*	+	+	+	−	+	−	−	−
Sal37	chicken	TE	*C*	+	+	+	−	−	−	+	−
Sal38	chicken	−	*C*	+	+	+	−	−	−		−
Sal39	chicken	AMP, TE, and SXT	*B*	+	+	+	−	+	−	+	−
Sal40	chicken	AMP, SAM, TE, and CIP	*C*	+	+	+	−	+	−	+	−
Sal42	chicken	−	*C*	+	+	+	−	−	−	−	−
Sal43	chicken	TE	*B*	+	+	+	−	−	−	+	−
Sal44	chicken	GN, TE, CIP, and SXT	*B*	+	+	+	−	+	−	+	−
Sal45	chicken	CIP and SXT	*E*	+	+	+	−	−	−	−	+
Sal46	chicken	AMP, TE, and SXT	*B*	+	+	+	−	+	−	−	−
Sal47	chicken	AMP and CIP	*C*	+	+	+	−	−	−	−	−
Sal48	chicken	−	*G*	+	+	+	−	−	−	−	−
Sal50	chicken	AMP, TE, and CIP	*E*	+	+	−	−	+	−	+	−
Sal52	chicken	TE	*C*	+	+	+	−	−	−	+	−
Sal53	chicken	TE and CIP	*C*	+	+	+	−	−	−	+	−
Sal54	chicken	CIP	*C*	+	+	+	−	−	−	+	−
Sal55	chicken	AMP and TE	*C*	+	+	+	−	+	−	+	−
Sal56	chicken	AMP, CTX, CRO, FEP, GN, TE, and CIP	*B*	+	+	+	−	+	−	−	−
Sal57	beef	−	*B*	+	+	−	−	−	−	−	−
Sal58	beef	−	*B*	+	+	−	−	−	−	−	−
Sal59	beef	−	*E*	+	+	−	−	−	−	−	−
Sal60	beef	−	*E*	+	+	−	−	−	−	−	−
Sal62	beef	−	*E*	+	+	−	−	−	−	−	−
Sal63	beef	−	*C*	+	+	−	−	−	−	−	−
	Number of isolates (%)			58 (100)	58 (100)	0 (0)	19 (32.76)	0 (0)	23 (39.66)	3 (5.17)	

+ represent as “present “; − represent as “not present”.

**Table 3:** The antibiotic resistance phenotypes of the *Salmonella* isolates.

Antimicrobial Agent	Number of Isolates Tested	Anti-Biogram Phenotypes of *Salmonella* Isolates Number of Isolates (%)		
		Sensitive	Intermediate	Resistant
**Group Penicillin**				
ampicillin (AMP)	58	32 (55.17)	0 (0)	26 (44.83)
**Group Combined β-lactam agents**				
ampicillin/sulbactam (SAM)	58	49 (84.49)	6 (10.34)	3 (5.17)
piperacillin/tazobactam (TZP)	58	56 (96.55)	2 (3.45)	0 (0)
**Group Cephalosporin**				
cefepime (FEP)	58	51 (87.93)	0 (0)	7 (12.07)
cefotaxime (CTX)	58	47 (81.03)	4 (6.90)	7 (12.07)
ceftazidime (CAZ)	58	52 (89.66)	5 (8.62)	1 (1.72)
ceftriaxone (CRO)	58	51 (87.93)	0 (0)	7 (12.07)
**Group Aminoglycoside**				
gentamicin (GN)	58	51 (87.93)	0 (0)	7 (12.07)
amikacin (AK)	58	58 (100)	0 (0)	0 (0)
**Group Carbapenem**				
ertapenem (ERT)	58	58 (100)	0 (0)	0 (0)
meropenem (MEM)	58	46 (79.11)	12 (20.89)	0 (0)
imipenem (IPM)	58	54 (93.10)	4 (6.90)	0 (0)
**Group Tetracycline**				
tetracycline (TE)	58	26 (44.83)	0 (0)	32 (55.17)
**Group Fluoroquinolone**				
ciprofloxacin (CIP)	58	4 (6.90)	34 (58.62)	20 (34.48)
**Group Folate pathway antagonist**				
trimethoprime/sulfamethoxazole (SXT)	58	46 (79.31)	0 (0)	12 (20.69)
**ESBL**	**Number of isolates tested**	**Number of positive isolates (%)**	**Number of negative isolates (%)**	
ceftazidime	58	7 (12.07)	51 (87.93)	
cefotaxime	58	7 (12.07)	51 (87.93)	

## Data Availability

Not applicable.
